# Examining select sociodemographic characteristics of sub-county geographies for public health surveillance

**DOI:** 10.1186/s12963-024-00352-y

**Published:** 2024-11-01

**Authors:** D. Aaron Vinson, Angela K. Werner

**Affiliations:** 1 Environmental Public Health Tracking Branch, National Center for Environmental Health, Hite Consulting, Inc, Centers for Disease Control and Prevention, Atlanta, GA USA; 2 Division of Environmental Health Science and Practice, National Center for Environmental Health, Centers for Disease Control and Prevention, 4770 Buford Highway NE S106-6, Atlanta, GA 30341 USA

**Keywords:** Sociodemographics, Census tract, Sub-county, Aggregation, Environmental health, Surveillance, Tracking

## Abstract

**Background:**

Mapping health outcomes related to environmental health hazards at the county level can lead to a simplification of risks experienced by populations in that county. The Centers for Disease Control and Prevention’s National Environmental Public Health Tracking Program has developed sub-county geographies that aggregate census tracts to allow for stable, minimally suppressed data to be displayed. This helps to highlight more local variation in environmental health outcomes and risk data. However, we wanted to understand whether the aggregation method used was aggregating sociodemographically similar or dissimilar areas with one another. This analysis attempts to explore whether the distributions of select people who may be at increased risk for exposure to environmental health hazards as identified by the Tracking Program are preserved in these sub-county geographies with the census tracts used as the foundation to create them.

**Methods:**

Mean values of three sociodemographic characteristics (persons aged 65 years and older, people from racial and ethnic minority groups, and population below the poverty level) for each sub-county geography in five states were calculated and placed into five break groups. Differences in break groups were determined and compared for each sub-county geography and census tract.

**Results:**

The sociodemographic characteristics among the census tracts and two aggregated sub-county geographies were similar. In some instances, census tracts with a low population or a highly skewed population (e.g., very high percentage of population aged 65 years and older) were aggregated with dissimilar census tracts out of necessity to meet the requirements set by the Tracking Program’s aggregation methodology. This pattern was detected in 2.41-6.59% of census tracts within the study area, depending on the sociodemographic variable and aggregation level.

**Conclusions:**

The Tracking Program’s sub-county aggregation methodology aggregates census tracts with similar characteristics. The two new sub-county geographies can serve as a potential option for health officials and policymakers to develop targeted interventions using finer resolution health outcome and environmental hazard data compared to coarser resolution county-level data.

## Background

Local data are critical for understanding environmental health outcomes. Viewing data across smaller geographic units, such as census tracts, can highlight variation in the geographic distribution of health effects related to environmental health risks and exposures, which may be masked by larger geographic areas, like counties [1]. Knowing which local areas experience elevated rates of negative health outcomes can help develop more targeted interventions [2]. The display of health data often includes choropleth maps, which are maps of polygons representing administrative units shaded to correspond to levels of disease incidence or another event. While this type of map is used frequently, a choropleth map has the potential to oversimplify the distribution of disease by communicating that the disease is evenly distributed in the shaded polygon or by showing abrupt changes in disease incidence at polygon borders [3]. This oversimplification can be more apparent as the size of polygons increases, for example, with state-level data. Additionally, as the size of the polygons decrease, there can be a reduction in ecological bias [4], which can alleviate the ecological fallacy [5].

Person-level or point data would have the least amount of ecological bias, but this type of data has its own issues in environmental health. Point-data must be geocoded, the process of using geographic information system (GIS) software to match an address to a street and address in a digitized reference map [6]. The process of geocoding addresses introduces inherent error to data that can affect rates of diseases when geocoding to any geographic boundary [6]. Addresses are more likely to be inaccurately geocoded when the address contains a rural ZIP code [7], or more broadly as population density decreases [8]. Rare disease rates in rural areas may be underestimated because of these factors of geocoding [9].

The National Environmental Public Health Tracking Program (Tracking Program) at the Centers for Disease Control and Prevention (CDC) began in 2002 to perform surveillance of risk factors and health effects associated with environmental hazards [10]. Most of the data that the Tracking Program currently collects is at the state or county level. However, over the last several years, the Tracking Program has been developing and refining its methodology related to the collection, geocoding, analysis, display, and communication of sub-county data [11]. This includes collection of standardized census tract-level data including hospitalizations, emergency department visits, and cancer rates. While having finer resolution data helps to better inform environmental public health, data at the census tract level also present two key challenges: confidentiality and statistical stability [12]. The Tracking Program has been developing a process to address these issues to increase the availability and accessibility of local environmental public health data at a sub-county level.

To address these challenges, the Tracking Program developed a process to aggregate census tracts to meet two minimum population thresholds, which were based on increasing the number of geographic units while decreasing instability and suppression [13]. This resulted in two geographic levels for aggregation — one with a minimum population of 5,000 persons and one with a minimum population of 20,000 persons. These two population thresholds were determined to be most appropriate for aggregation by Werner and Strosnider [13] out of nine total population thresholds tested by the Tracking Program, using 2010 Decennial Census data. In The Tracking Program’s methodology, zero-population census tracts are first separated out from populated census tracts designated by the 2010 Decennial Census. These populated census tracts were then aggregated using the Geographic Aggregation Tool (GAT) [14] with the position of centroids for each aggregated group determined through weighting by the constituent block group populations for the 5,000-person minimum geography and by the constituent census tract populations for the 20,000-person minimum geography. After scanning the input geographies, the GAT created a subset of tracts that did not meet the user-specified minimum on their own. This subset of tracts were ordered from greatest to least population, beginning with the area closest to the specified population threshold, and then the GAT merged with the nearest neighboring centroid. For example, if the population threshold was 5,000, then the GAT ordered areas not meeting the 5,000 person threshold, starting the merging process at areas with a population of 4,999. The new aggregated units were nested in their parent counties to maintain existing geographic hierarchies and did not cross any state boundaries.

A common concern during the development of these new sub-county geographies was whether census tracts were being aggregated with similar or dissimilar census tracts to form the new geographic unit. To answer this question, an analysis was performed on select sociodemographic characteristics of the sub-county geographies to assess how they compared to the census tracts used to create them. The results of this exploratory analysis are presented here.

## Methods

### Data

Five states were selected for this data analysis: Arizona, Colorado, Florida, Maine, and New York. All are Tracking Program recipients who participated in the sub-county geographic aggregation piloting through the Tracking Content Workgroup, whose mission is to facilitate collaboration between Tracking Program members and partners. These states were selected as they include a range of characteristics such as total population, population density, and population demographics. Using 5-year census tract estimates from the 2010 American Community Survey, three sociodemographic variables were selected to represent potential people who may be at increased risk for environmental health hazards [15, 16]: persons aged 65 years and older; all non-white including Hispanic population (referred to as people from racial and ethnic minority groups hereon); and population below the poverty level. Five-year estimates for total census tract population were obtained from the same data source to use as the denominator for all census tracts within the study area [16]. 5-year Variance Replicate Estimate Tables for each variable in each state in the study area were obtained from the American Community Survey [17], using 2010–2014 estimates due to being the data year most near to 2010 available.

### Methodology

In each state, the proportion of the total population of persons aged 65 years and older was calculated for each census tract. Then, for the 5,000-person minimum geographic units and the 20,000-person minimum geographic units, the proportion of persons aged 65 years and older in these geographic units was calculated by taking the mean population proportion value of the census tracts within each of the aggregated sub-county geographies. More details on the Tracking Program’s aggregation methodology can be found in Werner and Strosnider [13]. By using the mean of the census tract population proportions, the communities of each census tract are preserved in the mean proportion values of the aggregated geographic levels. The value of each sociodemographic variable for each of the three geographic levels were then classified by Jenks natural breaks into five groups using the classInt R package [18]. When planning the analysis, different methods to classify the data were considered, including equal intervals and quantiles. Jenks natural breaks aims to minimize differences within classes while maximizing differences between classes [19]. Reviewing the sociodemographic variables used in this analysis indicated that the distributions were often skewed and contained outliers. Due to these characteristics of the data, the Jenks natural breaks method was used to classify the data in the analysis.

The number of classifications that each census tract shifted in each aggregated sub-county geography compared to its original classification was then calculated by subtracting the higher-level geography’s classification from the lower-level geography’s classification. For example, a census tract in Florida’s initial proportion of persons aged 65 years and older was classified into the third break group. In the 5,000-person minimum geography, the persons aged 65 years and older proportion was classified into the first break group. This census tract’s value in the analysis was classified as two. These values are what served as the basis of the analysis. A dissimilarity in aggregation was considered for census tracts that had a classification value change of two or more classifications. As some degree of change is expected due to the nature of aggregating smaller areas into larger ones, census tracts with a classification value change of one or zero were considered to be aggregating with similar census tracts. The same methods were then applied to the additional selected variables: (1) people from racial and ethnic minority groups, and (2) population below the poverty level.

For each demographic variable, margins of error were calculated for each state within the study area using methodology outlined by the Census Bureau [17] and the 2010–2014 5-year Variance Replicate Estimate Tables. These tables provide eighty “pseudo-estimates” that are used to calculate the variance of the official estimate. Using these replicates, new margins of error were calculated for the Tracking Program’s two aggregated geographic areas for the three variables of the five states within the study area. These new margins of error were compared to the official 5-year census tract margins of error published by the 2014 American Community Survey.

In order to better understand the data, a number of visualizations were generated using the ggplot2 function package [20] in R [21]. Histograms and boxplots were created for each sociodemographic variable in each state, comparing the distribution of the variable at each of the three geographies. Each census tract in the study area was then plotted on a set of scatter plots to show the relationship of the proportion of population of each sociodemographic variable at the census tract or 5,000-person minimum geography with the larger geography The total number of census tracts in each state that changed by two or more classifications for each study variable was calculated to determine how often census tracts were being grouped with dissimilar census tracts. Extreme outlier census tracts were then mapped using ArcMap 10.6.1 [22] to determine if any shared characteristics could be identified and whether the methodology needed to be refined. After review, no further refinements were considered.

## Results

**Fig. 1:**
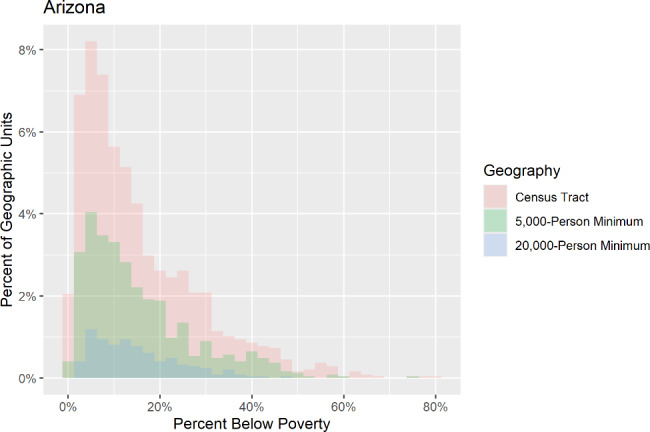
Proportions of population below the poverty level of the three sub-county geographies in Arizona

**Fig. 2:**
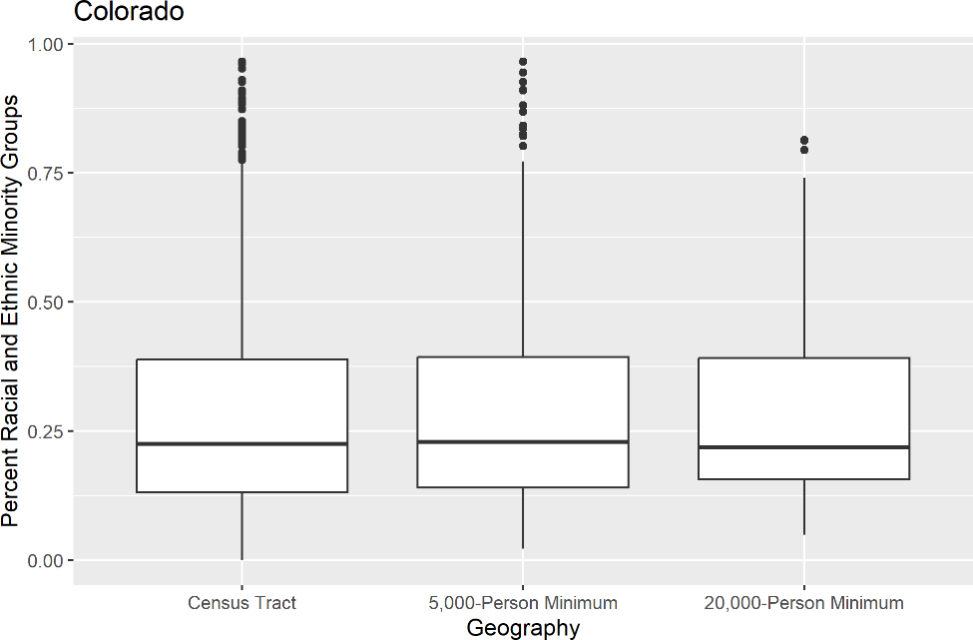
Proportions of people from racial and ethnic minority groups of the three sub-county geographies in Colorado

After examining the range of values across the three aggregation levels used in the study of the proportion of each sociodemographic variable, histograms showed that the range of proportions was generally maintained with similar distributions across the geographies. Figure 1 shows an example of the distribution of the below poverty population amongst the three geographies in Arizona, with each geographic level displaying the same general trend in distribution. The farthest outlier in Arizona for the proportion of population below the poverty level at the census tract level was 0.81, 0.74 at the 5,000-person minimum geography, and 0.49 at the 20,000-person minimum geography. Additionally, box plots showed that each geography had similar quartiles and median values for each sociodemographic variable in each state, though some loss of extreme values was shown in the higher-level geographies. Figure 2 shows an example of the quartile and median values for the proportion of people from racial and ethnic minority groups amongst the three geographies in Colorado.

**Fig. 3:**
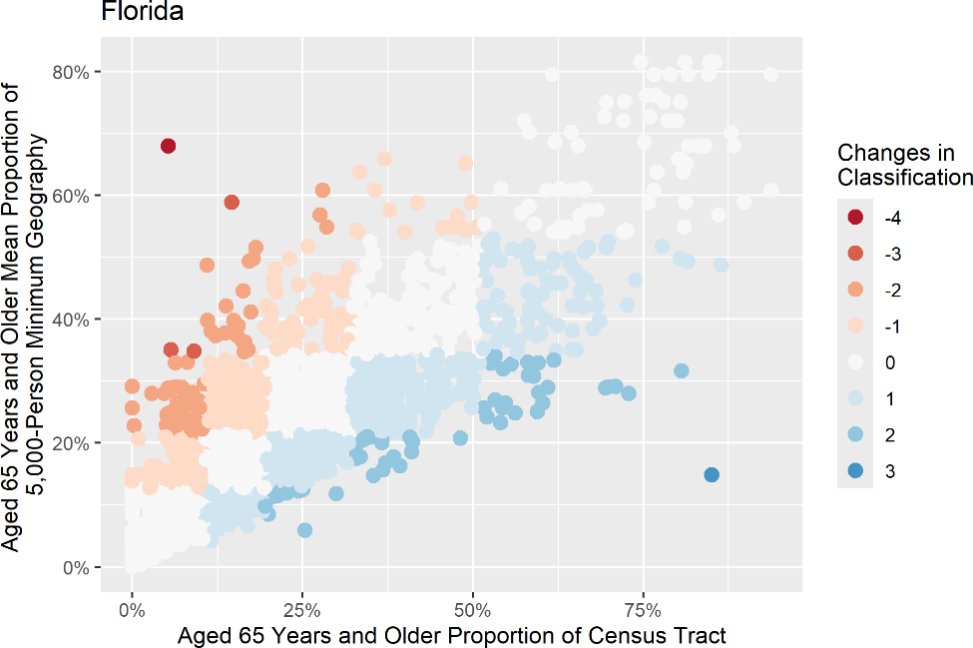
Scatter plot of persons aged 65 years and older in census tracts and 5,000-person minimum geographies in Florida

**Fig. 4:**
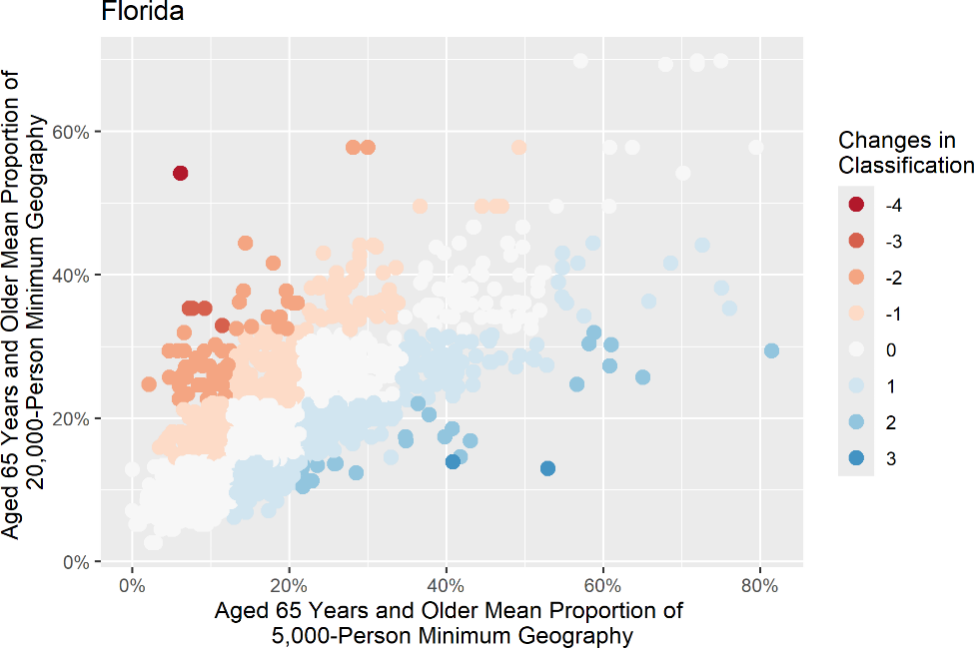
Scatter plot of persons aged 65 years and older in 5,000-person minimum and 20,000-person minimum geographies in Florida

Two sets of scatter plots were generated to display the change in median values of each study variable for census tracts that changed by two or more classifications in each state. In the first set, the proportion of each study variable of the census tract is along the X-axis, and the mean proportion of the 5,000-person minimum geography is along the Y-axis. Figure 3 shows an example of this type of plot, displaying the changes in Florida’s population of persons aged 65 years and older. The second set plotted the mean proportion of each variable for each census tract at the 5,000-person minimum geography along the X-axis and the mean proportion of the 20,000-person minimum geography along the Y-axis. Figure 4 shows an example of this second set of scatter plots, displaying the changes in Florida’s population of persons aged 65 years and older at the second aggregation level. The scatter plots visualize how the proportion of each study variable changes for each census tract as it is aggregated.

These scatter plots were used to identify census tracts with large changes in median values between each geographic level for further examination. When the subset of outlier census tracts was reviewed, it showed that these census tracts shared similar characteristics of being lower in population with a high proportion of population in the study variable. For example, a census tract near the Miami, Florida area was found to move by three classification changes at the 5,000-person minimum geography compared to its base census tract proportion of persons aged 65 years and older. According to our data, this census tract contained a population of 20 persons, 85% of which were aged 65 years or older. Census tracts with this low of a population must be aggregated with additional neighboring census tracts to meet the minimum population threshold for an aggregated geography, largely reducing the proportion of the census tract’s original sub-population.

The study area (comprising the five states selected) contained a total of 12,292 census tracts. The number of census tracts that were determined to change by two or more classifications when aggregating from the census tract to 5,000-person minimum geography was 465 (3.78%) based on persons aged 65 years and older proportion, 297 (2.41%) based on people from racial and ethnic minority groups, and 499 (4.06%) based on population below-poverty-level proportion. When aggregating from the 5,000-person minimum geography to the 20,000-person minimum geography, 689 (5.61%) census tracts changed by two or more classifications for persons aged 65 years and older proportion, 454 (3.7%) census tracts based on people from racial and ethnic minority groups proportion, and 810 (6.59%) census tracts based on population below-poverty-level proportion. By comparing the percentage of census tracts in a state that changed by two or more classifications when aggregating, some outliers were identified. For example, 5.31% of census tracts in Maine changed by two or more classifications when aggregating to the 5,000-person minimum geography based on the proportion of people from racial and ethnic minority groups. Another instance of an elevated percentage of census tracts that changed by two or more classifications when aggregating to the 20,000-person minimum geography occurred in Maine’s persons aged 65 years and older. In that case, 12.57% of census tracts changed by two or more classifications with aggregation. Table 1 presents a summary table that further presents these results.

The findings of the analysis as presented in Table 1 show that the aggregation methodology performed similarly in the study area for the selected sociodemographic characteristics. The percent of the total census tracts that change by two or more classifications in the census tract to 5,000-person minimum geography aggregation step and the 5,000-person minimum geography to the 20,000-person minimum geography aggregation step is within reason of each other across each state in the study area for each sociodemographic characteristic, with some previously noted exceptions in specific states for specific sociodemographic characteristics. Within reason was determined by looking at the overall effect across the study area and variables and are presented in Table 1. With these results, the Tracking Program concluded that its aggregation methodology does aggregate similar census tracts with others to create the new sub-county geographic units.

**Table 1:** Census tracts that changed by three or more classifications across all geography levels

	AZ	CO	FL	ME	NY	Total
**Total number of census tracts (N)**	1522	1249	4243	358	4920	**12,292**
% of total	12.38%	10.16%	34.52%	2.91%	40.03%	
**Census tract to 5**,**000-person minimum geography**						
**Aged 65 years and older**	45	47	130	13	230	**465**
% in state^b^	2.96%	3.76%	3.06%	3.63%	4.67%	
% of sociodemographic characteristic^c^	9.68%	10.11%	27.96%	2.80%	49.46%	
**Racial and ethnic minority groups**	45	47	130	13	230	**465**
% in state	2.96%	3.76%	3.06%	3.63%	4.67%	
% of sociodemographic characteristic	9.68%	10.11%	27.96%	2.80%	49.46%	
**Below poverty**	45	47	130	13	230	**465**
% in state	2.96%	3.76%	3.06%	3.63%	4.67%	
% of sociodemographic characteristic	9.68%	10.11%	27.96%	2.80%	49.46%	
**5**,**000-person minimum geography to 20**,**000-person minimum geography**						
**Aged 65 years and older**	49	144	131	45	320	**689**
% in state	3.22%	11.53%	3.09%	12.57%	6.50%	
% of sociodemographic characteristic	7.11%	20.90%	19.01%	6.53%	46.44%	
**Racial and ethnic minority groups**	61	49	157	24	163	**454**
% in state	4.01%	3.92%	3.70%	6.70%	3.31%	
% of sociodemographic characteristic	13.44%	10.79%	34.58%	5.29%	35.90%	
**Below poverty**	91	105	242	55	317	**810**
% in state	5.98%	8.41%	5.70%	15.36%	6.44%	
% of sociodemographic characteristic	11.23%	12.96%	29.88%	6.79%	39.14%	

^a^ This represents the number of census tracts in a state that changed by two or more classifications when moving geography levels.

^b^ The percent in state denotes the percentage of census tracts in the state that changed by two or more classifications when moving geography levels.

^c^ The percent of sociodemographic characteristic is the percent of all census tracts in the study area in each state that changed by two or more classifications when moving geography levels.

**Fig. 5:**
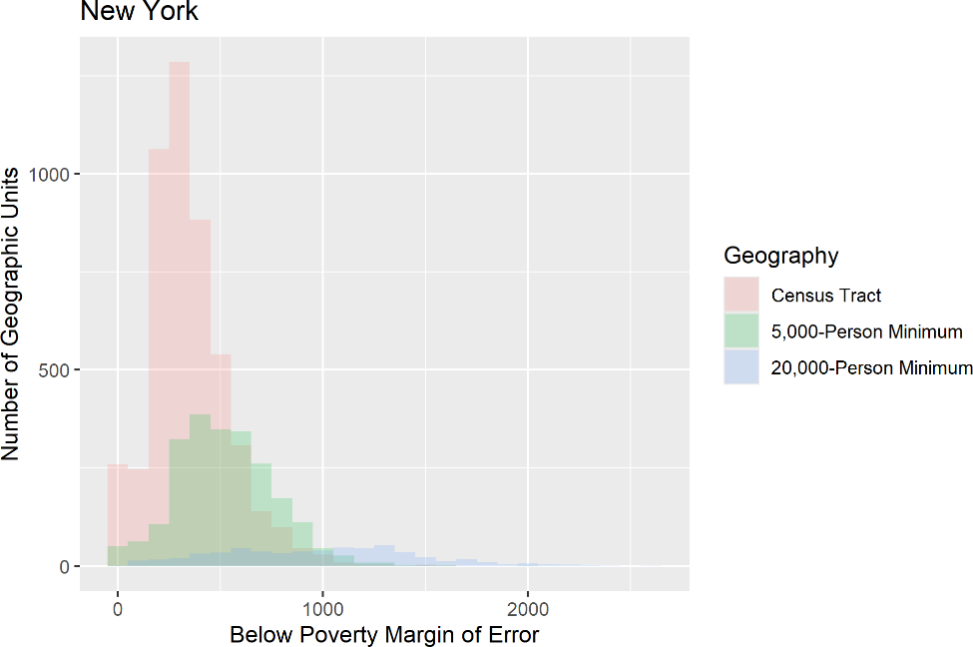
Histogram comparing margins of error for three sub-county geography levels for the population below poverty in New York

The calculated margins of error for the selected variables provided mixed results when reviewed. Margins of error (MOEs) for the population below poverty were consistently larger than the census tract MOEs, regardless of the state or geographic level. MOEs for population aged 65 and older were more narrow for both the 5,000-person minimum and the 20 − 000 person minimum geographic levels compared to the published census tract MOEs. Racial and ethnic minority MOEs at the 5,000-person minimum geographic level were more narrow than the published census tract MOEs for all states. Racial and ethnic minority MOEs at the 20,000-person minimum geographic level were more narrow compared to the census tract MOEs in Colorado, Florida, and Maine. Racial and ethnic minority MOEs at the 20,000-person minimum geographic level in New York and Arizona were somewhat wider compared to the published census tract MOEs. Figure 5 shows an example of the comparison done for these margins of error.

## Discussion

The aim of this study was to explore how proportions of sub-populations in census tracts changed as census tracts were aggregated to meet a minimum population threshold for the Tracking Program’s sub-county geographies. Generally, the three sociodemographic variables that were examined across aggregation levels (i.e., census tracts, 5,000-person minimum geography, and 20,000-person minimum geography) aligned with the distribution of these characteristics for populations at the census tract level for each state. However, some outliers were identified, particularly in states with low overall proportions of the sociodemographic variable being explored. For example, Maine was found to have many census tracts being aggregated with dissimilar census tracts when examining the people from racial and ethnic minority groups variable. According to the 2010 Census, Maine had a total white non-Hispanic population of 94.4% [23]. The margins of error calculated for The Tracking Program’s two geographic levels showed that the margins of error generally narrowed compared to the published census tract MOE in most combinations of variables, state, and geographic level. The widening of the MOEs for the population below poverty could be due to using a different denominator compared to the other variables. The Census Bureau does not determine poverty status for people living in group quarters, college dormitories, military barracks, unconventional housing living situations, and unrelated individuals under the age of 15. Due to this, the data calculated in this analysis for this variable were not the total population as it is for the other two variables examined. Due to the nature of some of these exemptions, a noticeable difference in population for whom poverty status is determined may be seen compared to the total population such as in the case of college dormitories or military barracks.

The outlier census tracts were identified using the scatter plots. Afterward, a follow-up examination of a sample of the outlier census tracts was performed to identify any key characteristics that might keep a census tract from aggregating with similar census tracts. One example of this was a census tract outside of the Miami, Florida area, which had a magnitude of three classification changes in the mean proportion of persons aged 65 years and older. This means that its 5,000-person minimum geography aggregated mean proportion of persons aged 65 years and older was much lower than the mean proportion for the same variable in the census tract. An examination revealed that this census tract had a total population of 20 people, 85% of which were aged 65 years and older. Utilizing the Tracking Network’s methodology for the 5,000-person minimum geography, this census tract had to combine with 4,980 residents in neighboring census tracts to become a new sub-county geography. Contributing a small amount of the required 5,000 population inherently lowered this census tract’s influence on the demographics of the aggregated geography. Based on our analysis, it is rare for a census tract with such a skewed proportion of population, such as the one identified, to be neighbored by census tracts with equally skewed proportions.

Out of necessity, these census tracts will have to be aggregated with census tracts with dissimilar sociodemographic characteristics. This effect was seen in several other areas, including Buffalo, New York and Tucson, Arizona. While low population census tracts can be found across the country for many reasons, this effect may be seen more frequently in areas where lower population census tracts are more common such as the Western region of the United States. The Census Bureau defines census tracts as having an optimum size of 4,000 residents [24]. Because of a lower population density and lack of roads or water features to serve as natural barriers, the spatial size of census blocks, and by extension census tracts, increases in the Western region to meet this optimum population size [25].

Small-area public health datasets have many potential applications, with several state and local health departments developing proprietary geographies to suit their needs [26]. These methods have the potential to involve methodology that is not translatable for other areas to incorporate such as neighborhood boundaries or data specific to a jurisdiction. Alternatively, the Tracking Program’s methodology uses national datasets for its aggregation parameters, with sub-county boundaries generated for all 50 states and available for public use [13]. This potentially reduces the burden on state and local health departments for creating their own small-area public health datasets and can be especially useful for communities of underserved populations that may be underrepresented in typical county or regional data [27]. These populations are often too small to calculate demographically stratified stable rates using the available data. Spatial modeling methods, such as Bayesian modeling, use available observations to interpolate estimates into areas where data are unavailable or improve existing data with few observations [28]. The Tracking Program’s aggregation boundaries can highlight the geographic location of communities with few or unavailable data with a statistically stable total population without the need of modeling or estimating data. The Tracking Program’s approach to addressing these issues in small area data could avoid some drawbacks of Bayesian modeling, such as low precision where the aggregation of census tracts may be needed to improve or an increase in temporal aggregation [29].

Some limitations are present in this analysis. The data are 5-year census tract estimates produced by the 2010 American Community Survey, while the sub-county geographies were aggregated using populations from the 2010 Decennial Census. This could potentially produce some variance in the conclusions of the analysis. The analysis also lacks an inferential statistical method to determine if there is a statistical difference in the sociodemographic variables at each geographic level, leaving only descriptive statistics used in the analysis. The analysis is limited in its scope. The study area was chosen to represent different types of states in geographic size and population size; it does not account for all 50 states of the United States. Additionally, while the sociodemographic variables chosen are populations at elevated risk to environmental health outcomes [30, 31], the variables are broad, limited to three measures, and do not illustrate all people who may be at increased risk for environmental health hazards; rather, these were intended to better understand how the sociodemographics were similar or dissimilar across the sub-county geographies. Considering these limitations, the Tracking Program concludes that these sub-county geographies are useful to serve as suitable boundaries for health, community, and environmental data to be used in applied fields of public health.

Future steps include expanding the scatter plot analysis to all census tracts in the United States to further explore trends or patterns in census tracts that may change by two or more classifications when aggregated to the two population thresholds. The Tracking Program is also exploring a third standardized geography — a 50,000-person minimum population geography to aggregate census tracts and counties – to allow for displaying rarer health outcomes (in the context of the Tracking Program) and other data.

## Conclusions

Overall, the analysis presented in this paper showed that the Tracking Program’s methodology to aggregate census tracts to create sub-county geographies that meet a minimum population threshold maintains the distributions of the selected sociodemographic characteristics of the census tracts, except in rare cases. The effect of the aggregation process on preserving the distribution of sociodemographic characteristics not explored in this analysis remains undetermined. The two sub-county geography types, with 5,000- and 20,000-person minimum populations, can enhance the Tracking Program’s ability to display and visualize environmental health outcomes at a more granular resolution than the county level, offering health officials and policymakers more detailed information to better target populations.

## Data Availability

The datasets used and analyzed during the current study are available from the corresponding author on reasonable request.

## Abbreviations

CDC: Centers for Disease Control and Prevention
Tracking Program: National Environmental Public Health Tracking Program
